# The avian cell line AGE1.CR.pIX characterized by metabolic flux analysis

**DOI:** 10.1186/1472-6750-14-72

**Published:** 2014-07-30

**Authors:** Verena Lohr, Oliver Hädicke, Yvonne Genzel, Ingo Jordan, Heino Büntemeyer, Steffen Klamt, Udo Reichl

**Affiliations:** 1 Max Planck Institute for Dynamics of Complex Technical Systems, Sandtorstr. 1, 39106 Magdeburg, Germany; 2 Current address: ProBioGen AG, Goethestr. 54, 13086 Berlin, Germany; 3 Cell Culture Technology, Bielefeld University, Universitätsstr. 25, 33615 Bielefeld, Germany; 4 Bioprocess Engineering, Otto von Guericke University Magdeburg, Universitätsplatz 2, 39106 Magdeburg, Germany

**Keywords:** Avian cell line AGE1.CR.pIX, Biomass composition, Flux variability analysis, Metabolic network modeling, Glutaminolysis

## Abstract

**Background:**

In human vaccine manufacturing some pathogens such as Modified Vaccinia Virus Ankara, measles, mumps virus as well as influenza viruses are still produced on primary material derived from embryonated chicken eggs. Processes depending on primary cell culture, however, are difficult to adapt to modern vaccine production. Therefore, we derived previously a continuous suspension cell line, AGE1.CR.pIX, from muscovy duck and established chemically-defined media for virus propagation.

**Results:**

To better understand vaccine production processes, we developed a stoichiometric model of the central metabolism of AGE1.CR.pIX cells and applied flux variability and metabolic flux analysis. Results were compared to literature dealing with mammalian and insect cell culture metabolism focusing on the question whether cultured avian cells differ in metabolism. Qualitatively, the observed flux distribution of this avian cell line was similar to distributions found for mammalian cell lines (e.g. CHO, MDCK cells). In particular, glucose was catabolized inefficiently and glycolysis and TCA cycle seem to be only weakly connected.

**Conclusions:**

A distinguishing feature of the avian cell line is that glutaminolysis plays only a minor role in energy generation and production of precursors, resulting in low extracellular ammonia concentrations. This metabolic flux study is the first for a continuous avian cell line. It provides a basis for further metabolic analyses to exploit the biotechnological potential of avian and vertebrate cell lines and to develop specific optimized cell culture processes, e.g. vaccine production processes.

## Background

Novel therapeutic options have been made possible with production of animal cell culture-derived biopharmaceuticals. An enormous amount of insight has been published on production of recombinant proteins (including antibodies) since the first products have been licensed for application in humans 1986 and 1987. CHO cells are the main substrate for recombinant proteins and therefore most metabolic studies describe these cells [1,2]. Regarding vaccine manufacturing, literature on properties and metabolism of host cells during growth and virus replication as well as process design and optimization is less abundant and not as focused. One reason is that safety considerations and execution of pre-clinical and clinical trials are paramount for vaccines because this medication usually is administered to healthy recipients. Another reason is the multitude of process options and host cell systems used for propagation of pathogens. For historic reasons, primary cultures (today usually embryonated chicken eggs or chicken embryo fibroblasts), diploid cell strains (human embryonic WI-38 and MRC-5), and few continuous cell lines (i.e. macaque-derived Vero cells or MDCK cells) are associated with the lowest possible risks to the vaccine recipients. Production with such cellular substrates often is performed in the presence of calf serum and adherent culture with little room for optimization. Furthermore, standardization of experiments is difficult with primary chicken material.

Several virus strains and viral vectors, e.g. Modified Vaccinia Virus Ankara (MVA), only replicate efficiently in avian cells [3]. Accordingly, primary chicken fibroblasts are still a commonly accepted production substrate. As vectored vaccines are gaining increasing importance [4], the potential of continuous suspension cells that proliferate in chemically-defined media are attracting more and more attention. Therefore, continuous avian cell lines are being investigated as a viable option to replace primary material. One possible new cell candidate that meets these criteria is the avian designer cell line AGE1.CR.pIX (in the following: CR.pIX) that proliferates in fully scalable suspension culture and is adapted to growth in a chemically-defined medium [5,6]. The latter property is an advantage for metabolic flux analysis as no unknown or complex components such as animal sera or hydrolysates that complicate carbon and nitrogen balance closure are present.

Metabolic models have been developed for a variety of cell lines to study physiological states or changes in metabolism as a response to different cultivation strategies, medium composition or stimuli like virus infection or accumulation of toxins. For example, the human cell lines HEK293 [7,8] and AGE1.HN [9,10], other mammalian cells like CHO [11], BHK [12], MDCK [13,14] and hybridoma cells [15,16] or insect cell lines like Sf9 [17,18] have been studied because of their relevance for the production of biopharmaceuticals. For all these cell lines, the two main pathways for energy generation and precursor supply were found to be glucose catabolism via glycolysis and glutamine catabolism via the TCA cycle, referred to as glutaminolysis [19-21]. Therefore, cell culture media usually contain substantial amounts of both substrates. Associated with an overflow metabolism based on glucose and a high glutaminolysis activity is the accumulation of lactate and ammonia in the cultivation broth with a negative impact on cell growth and product formation [22,23].

**Figure 1:**
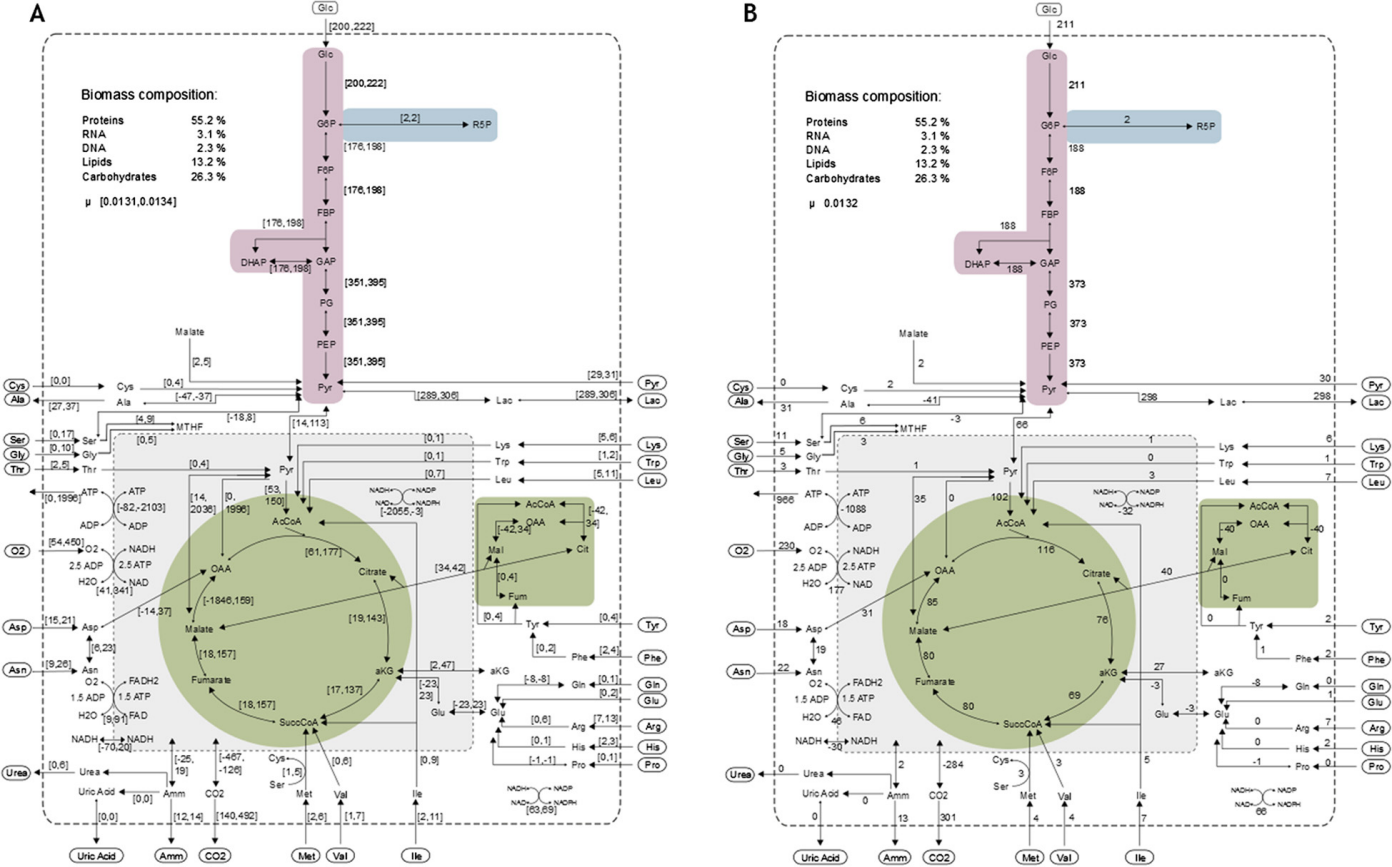
**Metabolic network model of the central metabolism of avian CR.pIX cells.** Main parts are glycolysis (purple), amino acid catabolism and the TCA cycle (green) which takes place in the mitochondria (grey). Reaction reversibility indicated by arrow heads. All fluxes are given in [μmol/gDW/h]. **A**: shows calculated flux ranges from scenario 1 (by FVA) in square brackets, **B**: shows determined rate values from scenario 2 (by MFA). The values are also given in Additional file 1: Table S2.

To describe cell metabolism, various mathematical methods have been developed and comprehensive reviews on applied approaches and their benefits for mammalian cell culture research are available [24,25]. Mass balancing techniques like metabolic flux analysis (MFA) can be performed for analysis of cellular growth, requiring only a comparatively small set of experimental data. During the exponential growth phase of batch cultivations or continuous cultivations this method can be used to evaluate possible intracellular flux distributions based on measurements of exchange fluxes. However, if the system is underdetermined, no unique flux distribution can be calculated. One approach to circumvent this limitation is to perform ^13^C labelling experiments. The higher experimental effort of such studies comes with the advantage of an increased number of constraints and uniquely resolvable flux distributions [26]. Another possibility is to apply flux variability analysis (FVA), a method related to MFA that calculates a flux range instead of a distinct flux value [27]. The advantage of this method is that it is experimentally less demanding as it requires (as MFA) only extracellular metabolite and cell concentrations as well as the biomass composition. Intracellular flux ranges from measured extracellular rates (uptake and release of metabolites) can then be computed with the help of software tools such as the *CellNetAnalyzer* toolbox [28].As a starting point for studying metabolism of avian CR.pIX cells during growth, we developed a network model for the central metabolism oriented in size and scope on other published models of the central metabolism of mammalian and insect cell lines (see Figure 1). Pathways were selected based on entries from avian species in the Kyoto Encyclopedia of Genes and Genomes (KEGG) database. As it was not known whether biomass composition of avian cells is comparable to other examined cells, we first determined relative proportions of biomass components experimentally. FVA and MFA were then applied to assess the metabolic behaviour of CR.pIX cells during exponential growth in a 1 L stirred tank reactor (STR). Finally, to verify emerging hypotheses regarding metabolic pathways, especially glutaminolysis, enzyme activity measurements and additional growth experiments were performed.

## Results and discussion

### Biomass composition

To allow metabolic flux analysis, some cell characteristics need to be known, i.e. specific dry cell weight and biomass composition. As it was not known whether similar results to other eukaryotic cells could be expected for an avian cell, most characteristics were determined experimentally rather than presumed.

The specific cell dry weight of CR.pIX cells was measured with 314 pg/cell. Amounts of DNA (2.3 ± 0.5% of biomass) and RNA (3.1 ± 0.2%) per CR.pIX cell were comparable to published values obtained for other eukaryotic cells. The protein content (55.2 ± 8.4%) was lower in CR.pIX cells than the typical range of 70–75% determined for other cells [7,16,29]. However, there are reports supporting a low protein content such as Zupke et al. who found 60% protein in mouse hybridoma cells and Carnicer et al. who measured a protein content of only 37% for yeast cells [30,31]. The relative amounts of amino acids of whole cell protein determined from CR.pIX cells are in general similar to published data on yeast [31], mammalian cells [9,29,32] and insect cell lines [33] (see Additional file 1: Table S1). The remaining fraction of biomass was assigned to lipids and carbohydrates in a ratio of 1:2. The estimated lipid content (13.1%) agrees with previous reports that vary between 9 and 20%, whereas the assumed carbohydrate content (26.3%) is at the upper limit of the wide range of 3.5–25.0% reported in literature [7,16,29,30,34]. However, a sensitivity analysis showed that absolute fractions of lipids and carbohydrates have only a minor impact on rate values of the calculated flux distributions (data not shown).

### Growth phases of CR.pIX cells in STR

**Figure 2:**
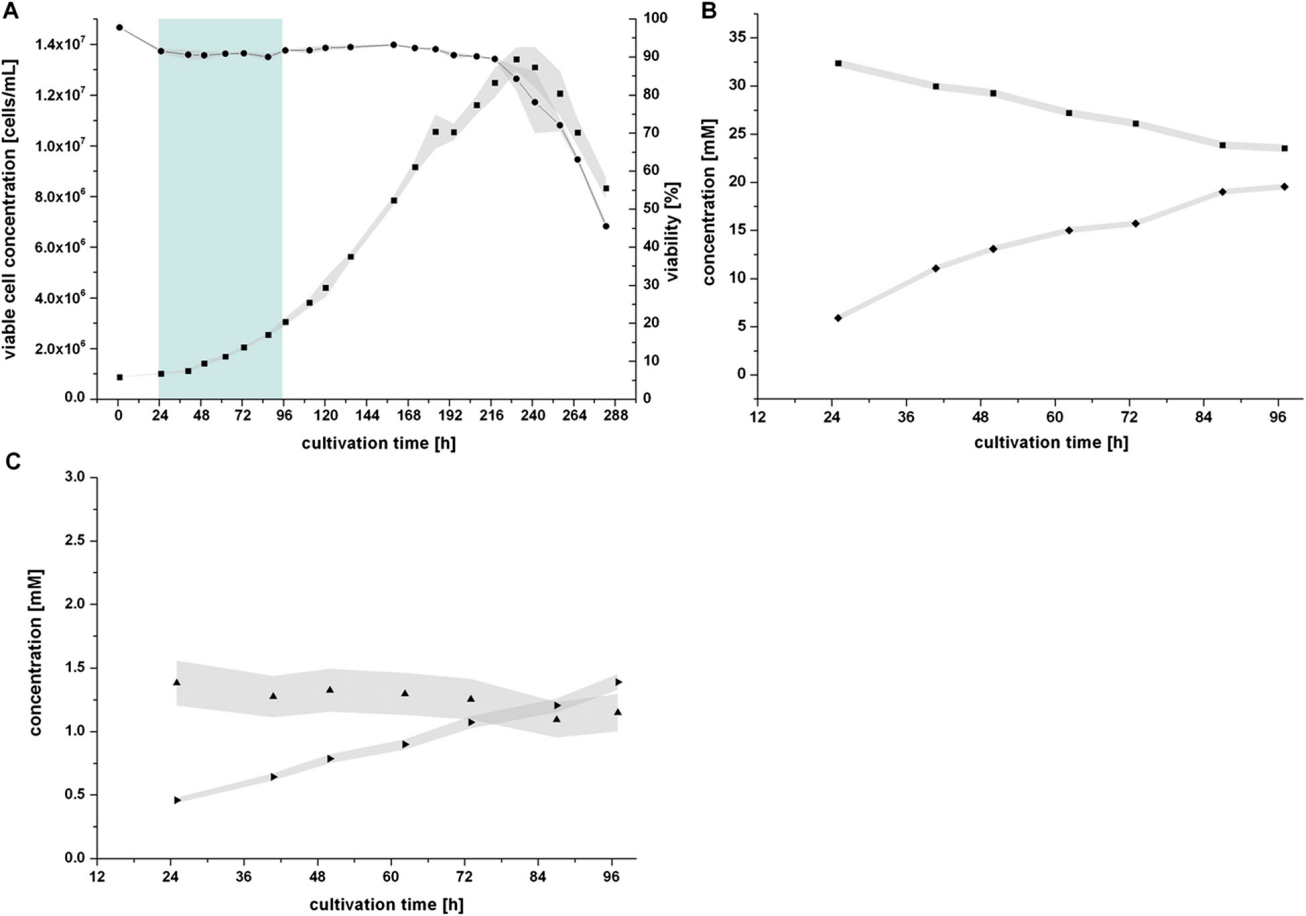
**Cultivation of CR.pIX cells in 1 L stirred tank reactor. A**: viable cell concentration (■) and viability (●) with the time frame used for MFA indicated by a pale blue box. Bands around data points indicate standard deviation from triplicate measurements. **B**: extracellular concentrations glucose (■) and lactate (♦); **C**: extracellular concentrations of glutamine (▲) and ammonia (►). Bands around data points indicate measurement errors from assay validations (see Additional file 1: Table S3).

The growth of CR.pIX cells can be divided into distinct phases. First, an initial lag phase was observed lasting for about 24 h with only slightly increasing viable cell concentrations. Thereafter, cells grew exponentially until 172 h. Following the exponential growth phase, a reduced growth was observed until 230 h, but viability was still above 90% in this intermediate growth phase (Figure 2A). After having reached the maximum viable cell concentration of 1.3 × 10^7^ cells/mL, cell death started.

The region chosen for MFA and FVA was 24–97 h, where a growth rate of 0.0132 h^-1^ was observed. In the following, only this time frame is considered when describing and discussing extracellular rates and intracellular flux distributions as it is very likely that the quasi steady state assumption holds. Furthermore, it is also the typical time frame in which cells are grown before infection in virus production processes (MVA or influenza A and B virus) [6,35]. Studies in this time frame are therefore most relevant in terms of vaccine manufacturing using CR.pIX cells.

### Exchange rates from measured extracellular metabolites

Determined exchange rates of CR.pIX were compared to rates given in other publications for mammalian cell lines (Table 1). We chose 6 data sets where equal units were given or could be calculated for comparison, thereof three publications on the metabolism of human AGE1.HN cells [9,10,36], one publication on HEK293 cells [7], and one on MDCK cells [13]. The majority of concentration profiles of CR.pIX cultivations are similar to concentration profiles obtained for other transformed cell lines studied in the past. For most of the metabolites, the measured exchange rates during CR.pIX cultivation have the same direction (uptake or release) and are in the same range compared to those found for other cell lines. Most notably, as observed for other cell lines, almost all of the consumed glucose is converted to lactate and excreted in the medium (Figure 2B). Key differences in the rates of CR.pIX cell cultivations were observed for the following metabolites:

**Table 1:** Determined extracellular fluxes for cultivation of CR.pIX cells in comparison with literature data

	**CR.pIX STR**	**AGE1.hn Shaker**^**a**^	**AGE1.hn Tube**^**b**^	**AGE1.hn Tube**^**c**^	**HEK293 STR**^**d,f**^	**MDCK STR**^**e,f**^
Time frame	24–97 h	18–90 h	72–169 h	0–74 h	0–72 h	16–80 h
Initial glc conc. [mM]	33	20–25	30	30	15	30
Initial gln conc. [mM]	1.5	5	2	4	1	2
Glc	-211.2 ± 10.8^g^	-123.0	-126.9	-429.2	-395.5	-1108.7
Lac	297.6 ± 8.8	263.3	87.6	639.8	439.0	2108.0
Pyr	-30.1 ± 0.8	-28.7	-2.9	-76.5	-	0.0
Gln	-0.1 ± 0.1	-37.1	-41.2	-95.5	-66.5	-70.9
Amm	12.6 ± 0.9	-	-	91.7	48.5	51.8
Glu	-1.4 ± 1.1	4.0	0.9	17.0	-1.4	-42.1
Ala	31.6 ± 4.9	17.5	8.0	20.6	11.8	-49.4
Arg	-9.9 ± 2.8	-8.2	-4.4	-12.4	-15.3	-11.7
Asn	-17.1 ± 8.5	-2.9	-2.0	-7.3	-4.6	-1.9
Asp	-17.8 ± 3.2	-14.1	-8.9	-16.2	-0.2	-2.0
Cys	0.0	0.2	-1.1	-0.2	-	-2.4
Gly	-4.8 ± 4.8	2.6	-0.8	6.8	17.0	2.3
His	-2.3 ± 0.4	-1.7	-0.7	-3.2	-3.5	-2.8
Ile	-6.2 ± 4.4	-8.1	-1.4	-14.7	-21.8	-12.3
Leu	-7.9 ± 3.2	-11.5	-3.2	-23.8	-24.7	-23.5
Lys	-5.5 ± 0.3	-6.9	-3.5	-16.6	-12.6	-22.9
Met	-4.1 ± 2.0	-3.1	-1.2	-6.6	-4.9	-6.4
Phe	-2.5 ± 1.0	-3.3	-1.5	-6.3	-5.4	-8.9
Pro	-0.1 ± 0.1	7.1	13.1	13.4	0.8	6.7
Ser	-8.3 ± 8.3	-12.2	-5.7	-33.4	-17.7	-16.8
Thr	-3.4 ± 1.9	-4.9	-2.0	-8.0	-9.5	-14.9
Trp	-1.5 ± 0.2	-0.9	-0.4	-0.8	-	-5.2
Tyr	-1.8 ± 1.8	-2.8	-1.4	-5.5	-4.6	-5.0
Val	-4.3 ± 2.8	-8.4	-1.8	-14.2	-19.1	-17.0
Uric acid	0.0	-	-	-	-	-

All rates are given in μmol/gDW/h.

^a^from [9]; ^b^from [36]; ^c^from [10]; ^d^from [7]; ^e^from [13,14].

^f^these fluxes were re-calculated to [μmol/gDW/h] with the given fluxes and cell dry weights.

^g^standard deviation from Monte-Carlo sampling using validated standard deviations of the methods as boundaries.

First, glutamine uptake is zero with CR.pIX cells (Figure 2C) as the observed glutamine concentration decrease is solely due to glutamine hydrolysis. Associated with this, only low amounts of ammonia accumulated in the medium (1.4 mM after 97 h: maximal 2.8 mM at the end of the batch cultivation at 210 h). For production of cell culture-derived products this is a beneficial characteristic as higher ammonia concentrations are known to be toxic with negative impact on cell growth and product formation [22]. The observed ammonia concentration is at the lower limit compared to other cells, where concentrations between 3 and 8 mM were reported to lead to impaired growth [37-39].

The second difference is observable for glycine which is taken up by CR.pIX cells, but released by other cell lines. This observation can be explained by the fact that glycine is reported to be an essential amino acid for birds and therefore needs to be provided for CR.pIX cells by the medium [40]. The third finding is that aspartate and asparagine are consumed by CR.pIX cells in higher amounts or at least at higher rates compared to other cell lines. Both amino acids can be converted to oxaloacetate and thus channeled into the TCA cycle. One could hypothesize that CR.pIX cells compensate in that way for the negligible glutamine uptake to fill the TCA cycle intermediate pools. We also observed that the measured uptake rates for the essential amino acids histidine, lysine and tryptophan were slightly below their minimal requirements for biomass formation with the determined growth rate. For the following analyses we therefore adjusted their upper boundaries slightly to fit the minimal stoichiometric demands for the measured specific growth rate.

### Intracellular flux distribution

Using a metabolic network model of the central metabolism of avian cells that we developed according to literature data and database entries, the metabolism of CR.pIX cells during cultivation in 1 L STR was studied using FVA (details see section “Flux variability and metabolic flux analysis”). The time span of 24 h and 97 h was chosen for analysis, as within this time interval during exponential growth, the quasi steady state assumption holds. FVA uses as few constraints for the metabolic network as possible in order to get an unbiased look at the metabolism. This generated a distribution of flux ranges that is named scenario 1 in this manuscript. However, flux ranges are sometimes difficult to interpret. Therefore, after having performed FVA, we set few reasonable constraints, e.g. uptake and excretion rates were set as fixed and the pyruvate carboxylase activity was assumed to be inactive. With these constraints a unique flux distribution could be calculated which is discussed as scenario 2 in the following.

All calculated flux ranges (scenario 1; determined by FVA) and fluxes (for scenario 2, determined by MFA) are given in the Additional file (Additional file 1: Table S2). Together with the network, main fluxes are also depicted in Figure 1. Although only flux ranges can be calculated with the constraints of scenario 1 (Figure 1A), some conclusions can already be drawn from them as we will discuss below. However, most of the following discussion will refer to the unique flux distribution calculated for scenario 2 (Figure 1B). With the applied constraints, the linear equation system had two degrees of redundancy. Therefore, the sum of the variance weighted squared residuals (*h*) need to be below 5.99 to exclude significant measurement errors (with an applied significance level of α = 0.05). Since we obtained an *h*-value of 0.63 we concluded that the model fitted the data sufficiently well.

#### Glycolysis and cytosolic pyruvate metabolism

Glucose is taken up with a high rate (211 μmol/gDW/h) and then processed via glycolytic reactions to pyruvate. Only 1% of the glucose is fed into the pentose phosphate pathway for nucleotide synthesis. Such low pentose phosphate influxes were also observed in other studies with transformed cells, e.g. 1.5% and 2.9% in human AGE1.HN cells [9,36]. The calculated ranges for the rates of glycolysis are relatively narrow as they mainly depend on the glucose uptake rate that was given by the measurement of extracellular glucose. Cytosolic pyruvate is mainly derived from glycolysis (92%); the rest of the cytoplasmic pyruvate is taken up by the cells and in small parts obtained from cysteine degradation and the malic enzyme reaction (together 1%). Similar to other transformed cells, cytosolic pyruvate is mainly converted to lactate (73%) or alanine (10%). Only a small fraction (16%) is transported to the mitochondria where it is converted to acetyl-CoA and further oxidized. However, if a complete pentose phosphate pathway (PPP) is included in the model, the exact ratio between glycolysis and PPP is unresolvable and the corresponding flux ranges would be notably wider. Since CO_2_ is produced via the PPP, a high PPP flux would reduce the amount of carbon that re-enters the lower part of glycolysis, and therefore even less pyruvate would enter the TCA cycle.

In general, the flux distribution at the cytosolic pyruvate node shows that the majority of the glucose-associated metabolism of CR.pIX cells is used to generate ATP and reducing equivalents via glycolysis. The known Warburg effect of enhanced aerobic glycolysis, leading mostly to lactate, can thus also be observed in transformed cells from avian origin. Furthermore, there seems to be a weak coupling to the mitochondrial TCA cycle as only a minority of the cytosolic pyruvate is channeled into the mitochondria. Other studies have shown that several transformed cell lines show low activities of the three pyruvate dependent enzymes pyruvate dehydrogenase (PDH), pyruvate decarboxylase (PC) and phosphoenolpyruvate carboxykinase (PEPCK) that can serve as connectors between glycolysis and TCA cycle [41,42]. For our cells, we calculated that the PDH (r33) must carry some flux (since the lower boundary of this reaction is strictly positive), but the non-resolvable PC reaction (r46) is not obligatory as its flux range also includes zero. Measurements on activities of anaplerotic enzymes will be discussed in a later subsection.

#### Mitochondrial pyruvate metabolism and TCA cycle

The mitochondrial pyruvate pool is fed from the cytosolic pyruvate pool (64%) and the anaplerotic malate conversion (34%). 100% of the efflux is catalyzed by the pyruvate dehydrogenase thereby generating 88% of the mitochondrial acetyl-CoA. Other influxes towards this TCA precursor come from amino acids, namely isoleucine (4%) and leucine (7%), tryptophan, and lysine (together 1%). The subsequent reactions of the TCA cycle are fed from other catabolic reactions: α-ketoglutarate from cytosolic influx and succinyl-CoA from isoleucine, valine, serine and methionine degradation. This shows the dependency of the TCA cycle activity on relatively high amino acid uptake and catabolic rates in CR.pIX cells.

#### Oxidative phosphorylation & ATP balance

We calculated high fluxes for the oxidation of NADH (177 μmol/gDW/h) and FADH_2_ (46 μmol/gDW/h). Since a transport reaction for NADH between the cytosol and mitochondria was included in the model, the pools of reducing equivalents for both compartments are balanced simultaneously. A minor flux for the corresponding transport reaction shifting reducing equivalents from the mitochondria to the cytosol was calculated with 30 μmol/gDW/h. By setting this transport reaction to zero, cytosolic and mitochondrial reducing equivalents have to be balanced separately. Besides an increased oxygen uptake and ATP synthesis rate, this leads to no significant differences in the flux distributions (data not shown).

ATP generation via the TCA cycle and the electron transport chain makes up to 74% of the total ATP generated. A smaller fraction (26%) is derived from glycolysis. This is similar to results from studies on mouse hybridoma and CHO cells [43,44] where values of 60–80% of ATP production via the TCA cycle have been reported. Although considerable ATP amounts are needed for lipid synthesis, amino acid transporters, upper glycolysis and biomass, the high (unspecific) ATP consumption through reaction r77 (966 μmol/gDW/h) indicates that there seems to be an overload with ATP which is either used for certain maintenance processes or passed through futile cycles.

#### Amino acid and nitrogen metabolism

Degradation rates of some amino acids, e.g. tryptophan and histidine, are low, indicating that these amino acids are taken up to match the anabolic demand for synthesizing biomass rather than to further metabolize them via catabolic routes. The calculated rate ranges for most fluxes of the amino acid catabolism are constrained to relatively small intervals (e.g. for degradation of valine and histidine). There is no uric acid synthesis (r68) as no uric acid could be measured experimentally. Although this reaction is known to take place in avian cells, this is not a fully unexpected finding. Uric acid was detected in livers and kidneys of chicken and turkey, but not in all bird tissues [40]. CR.pIX cells, as other transformed cells, use ammonia as a sink and release the excess cytosolic ammonia (that is produced by deamination reactions of amino acids) into the medium where it accumulates. However, due to low glutamine exploitation, the ammonium concentration stays at a low level so that toxic concentrations are not reached.

Interestingly, the fluxes through the glutamine synthetase (r48) (i.e. in the direction of glutamate synthesis) are negative as glutamine uptake was not sufficient for biomass synthesis and glutamine therefore has to be synthesized from the glutamate pool. Glutamate itself is likely to be mainly derived from degradation reactions of isoleucine, valine and aspartate. Especially aspartate is taken up in large parts (18 μmol/gDW/h) and can be converted to glutamate. Other amino acids that could compensate for glutamine conversion to glutamate are arginine, histidine and proline. However, only arginine is taken up in considerable amounts by the cells so that one could speculate that the path from glutamate to α-ketoglutarate is generally not used extensively by the CR.pIX cells and thus, the classical dependency on glutamine is not given for CR.pIX cells. To elucidate unambiguously the source of carbon for glutamate synthesis, ^13^C labelling experiments would be required, which are beyond the scope of the current contribution.

### *In vitro* enzyme activities

To validate the two hypotheses from the previous section that i) glycolysis is only weakly connected to the TCA, and that ii) glutaminolysis plays a minor role in energy supply and precursor generation, we measured the maximum *in vitro* enzyme activities of the related enzymes.

For the first hypothesis, we measured maximum activities of PC and PDH. For both reactions, low enzyme activities of 0.2 nmol/10^6^cells/min (i.e. 39 μmol/gDW/h) were measured as for MDCK cells [45]. These values supported the applied constraint of a missing carboxylating anaplerotic reaction from pyruvate towards oxaloacetate. However, the measured low PDH activity seems to contradict the calculated flux of 102 μmol/gDW/h. One possible explanation is given by the significantly smaller calculated lower boundary of this flux when applying fewer and less stringent assumptions with scenario 1. However, at least a basal activity is required as the lower boundary is strictly positive (53 μmol/gDW/h).

The second hypothesis, stating that glutaminolysis is inversed, was validated via measurements of glutaminase (converting glutamine to glutamate) and glutamine synthetase (converting glutamate to glutamine). Glutaminase showed a maximal activity of 0.1 nmol/10^6^cells/min (i.e. 19 μmol/gDW/h), which is an even lower value than the measured 1.5–7.4 nmol/10^6^cells/min for MDCK cells [45,46]. The flux from glutamate to glutamine was predicted by flux analysis to be active towards glutamine in order to account for the unmet demand of glutamine for biomass formation. The measured enzyme activity of the catalyzing enzyme glutamine synthetase showed a maximal activity of 5.9 nmol/10^6^cells/min (i.e. 1135 μmol/gDW/h). This value is on the one hand significantly higher than the value for the reverse reaction and on the other hand at the upper limit of reported values for MDCK cells (0.6–6.5 nmol/10^6^cells/min, [45,46]). Thus, although values from *in vitro* enzyme activity measurement and flux analysis are not directly convertible, the measured enzyme activities support the calculated flux distribution towards glutamine and the low level of glutamine usage.

### Growth in glutamine-free medium

**Figure 3:**
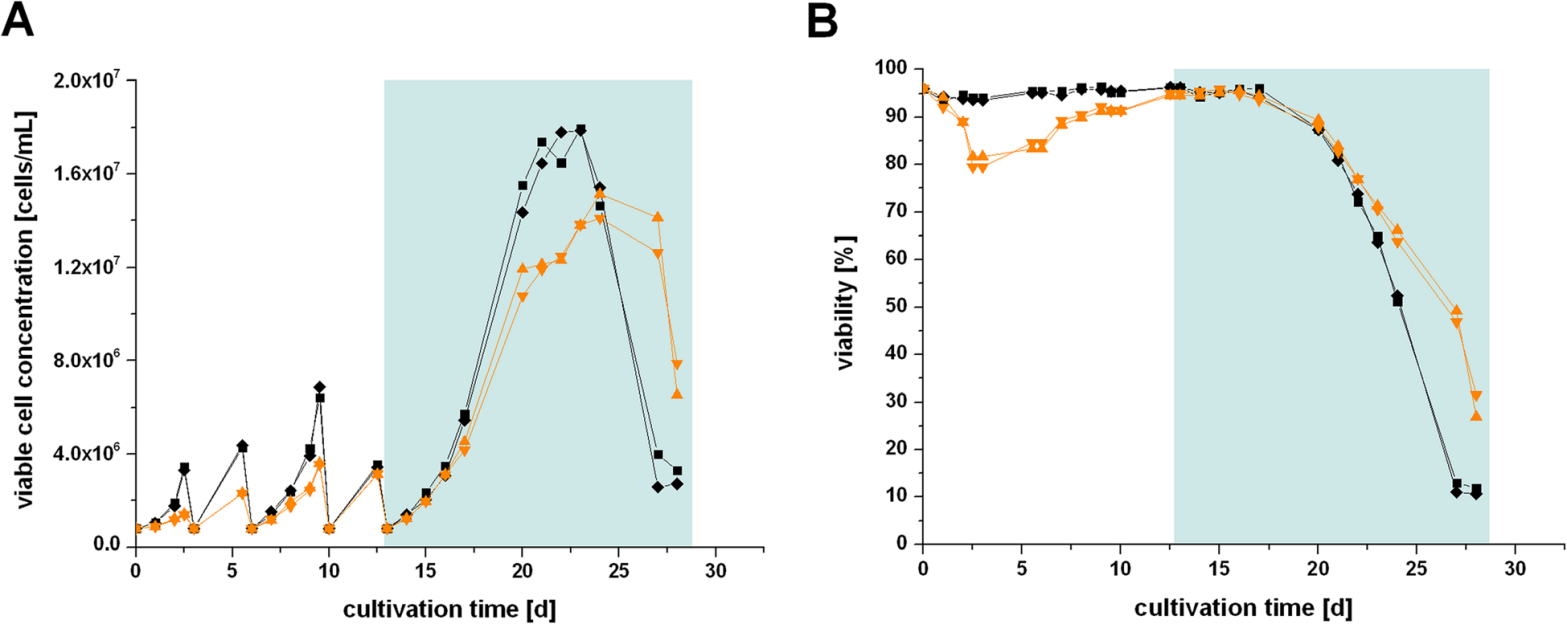
**Cultivation of CR.pIX cells with (black squares) or without glutamine (orange triangles) in shaker flasks (two parallel cultures each).** Viable cell concentrations **(A)** and viabilities **(B)** are shown for 5 subsequent passages of CR.pIX cells in the respective medium. The pale blue box highlights the 5^th^ passage for which a growth curve was monitored.

Since the uptake flux for glutamine was calculated as zero and the enzyme measurements supported the low glutaminolytic activity, we speculated whether glutamine is even dispensable for growth of CR.pIX cells. To validate this hypothesis, we performed shaker flask cultivations of CR.pIX cells in CD-U2 medium with 2 mM glutamine and in CD-U2 medium without glutamine addition. As no serum is present in the medium, other glutamine sources can be excluded. Cultures with or without glutamine were run in parallel over 5 passages. For passage 5, a growth curve was recorded. Figure 3 shows that cells without glutamine did not grow as well as in the presence of glutamine during the first three passages (Figure 3A). Viability also dropped below 90% in these cultures (Figure 3B). After passage 3, cell growth performance in glutamine-free medium increased significantly. Finally, cells in the 5^th^ passage in glutamine-free medium did grow comparably fast as in the presence of glutamine. Maximal viable cell concentrations of 1.4 × 10^7^ cells/mL were achieved with viabilities above 90%. This experiment demonstrated that CR.pIX cells are capable of glutamine-independent growth. With CHO cells, this was achieved after several selection rounds [47], but could not be achieved with other cells [48,49]. Considering that we did not measure glutamine uptake fluxes, we were surprised at the intermittent decrease in proliferation rates and viabilities of CR.pIX cultures after glutamine removal. However, extracellular and intracellular glutamine mediate also non-nutritional effects that can impact apoptosis and heat shock responses [50,51]. We hypothesize that the short adaptation period after glutamine removal may be due to shifts in glutamine-responsive signaling pathways. Infection experiments with poxvirus MVA showed that productivity of CR.pIX cells is not negatively affected by glutamine absence (data not shown). The feature of glutamine-free growth of CR.pIX cells may be advantageous in particular for high cell density cultivations were ammonia accumulates to very high concentrations in the culture supernatant and prevents further increases in cell numbers and productivity [38,52].

## Conclusions

Avian cells are important substrates for producing a number of licensed vaccines and various viral vector-based processes under development. The only avian substrates that are currently approved for production are embryonated chicken eggs or primary chicken embryo fibroblasts. Because processes using primary cultures are difficult to optimize and standardize, access to well characterized continuous avian cell lines, such as the CR.pIX cell line derived from a muscovy duck embryo, would be a significant step forward.

Here, we determined metabolic properties of the CR.pIX cell line to complement previous developments on scalable vaccine production processes in chemically defined media. The current study comprised three parts: i) analysis of biomass composition and measurements of cell and extracellular metabolite concentrations, ii) construction of a stoichiometric network for the central metabolism of avian cells and subsequent analysis with flux variability and metabolic flux analysis, and iii) experiments and analytics to validate hypotheses derived from the stoichiometric analysis.

The first part expands the available data base on the metabolism of immortalized cell lines. Results on biomass composition and growth properties of the avian CR.pIX cell line agree with data determined for other transformed mammalian and insect cell lines used in biopharmaceutical production.

Flux variability and metabolic flux analyses in the second part reveal an overflow metabolism similar to mammalian cells with high uptake of glucose followed by conversion to and release of lactate and alanine. The TCA cycle was only weakly connected to glycolysis and appeared to depend on the influx of amino acids, mainly isoleucine, valine and aspartate. Finally, our measurements together with flux (variability) analysis indicated that glutamine supplementation is not an essential requirement for CR.pIX cell proliferation.

The derived hypothesis on low glutaminolysis activity could be confirmed by enzyme activity measurements. However, the hypothesis of a weak connection between the cytosolic and mitochondrial pyruvate pools could not be confirmed entirely. To elucidate whether cytosolic pyruvate is transported in significant amounts from the cytosol to the mitochondria, e.g. ^13^C labelling experiments could be helpful, which was beyond the scope of the current contribution. Low dependence on glutamine was confirmed by passaging cells in glutamine-free medium and agrees well with the observation that ammonia is accumulating to very low concentrations in the medium. This is a very beneficial property of CR.pIX cells especially for fed-batch cultivation strategies where ammonia often accumulates to toxic levels.

Overall, the present study provides a basis for a more focused metabolic analysis of avian cell lines and thus helps to further optimize medium and feed requirements. Acute infection and associated take-over of the metabolism by a virus may shift the requirements for certain nutrients. The derived model may therefore also provide a context for future studies on the metabolism of CR.pIX cells during the virus replication phase.

## Methods

### Cell cultivations

CR.pIX suspension cells were routinely cultivated in 250 mL shaker flasks (working volume 150 mL, with membrane cap for aeration) in a chemically-defined medium (CD-U2, PAA) supplemented with glutamine (2 mM final concentration, Sigma), alanine (2 mM final concentration, Sigma) and a recombinant insulin-like growth factor (Long-R3 IGF, 10 ng/mL final concentration, Sigma). Flasks were incubated at 185 rpm with 5 cm amplitude, 37°C and 5% CO_2_. Pre-cultures for bioreactor experiments, cultures for determination of biomass composition as well as the comparative cultivations in glutamine-containing and glutamine-free medium were performed in shaker flasks. For inoculation of a 1 L stirred tank bioreactor (STR, DasGip AG, vessel: Spinner Type BS, cellferm-pro® system), 8 × 10^5^ cells/mL in 1 L CD-U2 medium were transferred into the bioreactor. The bioreactor was equipped with a pitched-blade stirrer that was operated at 120 rpm. The temperature was controlled at 37°C; pO_2_ was set to 50% by pulsed aeration with air enriched with variable contents of O_2_ and CO_2_. During the first 60 h, pH was slowly reduced from 7.4 (initial value of the medium) to 7.0 and controlled at this value subsequently by addition of CO_2_ or 1 M Na_2_CO_3_.

### Measurement of biomass composition

All assays and analytics were performed with cells in the exponential growth phase. Therefore, samples were taken from shaker flask cultures at 72 h post inoculation. For each analysis, 3 biological replicates were measured with at least 3 technical replicates each.

For determination of dry cell weight, cell suspension containing about 1 × 10^8^ cells/mL was centrifuged (500 × g, 10 min) and re-suspended in 10 mL of fresh medium. Samples were dried at 60°C until weight remained constant. The specific dry cell weight was calculated against weight of empty tubes and against a medium control (10 mL of fresh medium dried in tubes).

The BCA assay (Pierce/ThermoScientific) was used to determine the cellular protein content. Samples containing 4 × 10^6^ cells were centrifuged (500 × g, 10 min) and re-suspended in 1 mL lysis buffer (from CytoTox 96® Non-Radioactive Cytotoxicity Assay, Promega). After 15 min incubation at room temperature, samples were sonicated for 3 min in a water bath. Standard curves were prepared using BSA and analyzed together with samples by measuring the absorption at 562 nm (inifinite200 plate reader, TecanGroup).

For DNA purification, a culture volume containing 5 × 10^6^ cells was taken and purified with the QIAamp DNA Blood Mini kit (Qiagen) according to the procedure “protocol for cultured cells”. After purification, 2 μL of assay buffer (in which the samples were dissolved later) were pipetted onto 16 spots on a NanoQuantPlate (TecanGroup) as blank. After blanking, 2 μL of samples were measured. DNA concentration was then determined by multiplication of the measured absorption coefficient with 50 as a dsDNA specific factor. The same method as for DNA content analysis was employed for RNA content analysis. Here, purification was done with the NucleoSpin RNAII kit (Macherey-Nagel) according to the procedure “total RNA purification from cultured cells and tissue”. RNA concentration was determined from absorption measurements at 260 nm, but by multiplication with a factor of 40. Measurement procedure was analogous to the DNA analysis.

Amino acid contents were determined as follows: about 1 × 10^8^ cells were washed with 0.9% NaCl solution and re-suspended in 2 M HCl. Cells were incubated in acid at 100°C for 24 h. After this hydrolyzation, the solution was neutralized by adding 2 M NaOH and subsequently analysed by HPLC. Aspartate and glutamate percentages were corrected for a hydrolysis rate of 47.5% as described in literature [7].

A HPLC method was used to measure the amino acid composition of cell protein [53]. Due to harsh hydrolytic conditions, some amino acid concentrations could not be measured, but were estimated from literature values (see Additional file 1: Table S1).

### Cell and metabolite concentration measurements

Samples were taken once or twice a day. Cell concentration, viability and average cell diameter were determined using the Vi-CELL™ XR device (Beckman Coulter). Samples for measuring extracellular metabolite concentrations were centrifuged after sampling and the supernatant was stored at -80°C until analysis. Measurement of glucose, lactate and ammonia concentrations was done with a Bioprofile 100 Plus (Nova Biomedical). A HPLC method was used to measure the concentration of pyruvate. Extracellular amino acid concentrations for metabolic flux analysis were determined using a derivatization method and subsequent measurement in a HPLC system [54]. Uric acid concentration was measured with an enzymatic reaction assay kit (Uric Acid Assay Kit, BioVision) including standard curves and controls as described in the manual. Uric acid was only measured at culture start and end. Standard deviations for all concentration measurements (determined by method validations) can be found in Additional file 1: Table S3.

### *In vitro* determination of maximum enzyme activities

Maximum activities of pyruvate carboxylase (PC), pyruvate dehydrogenase (PDH), glutamine synthetase (GS) and glutaminase (GLNase) in exponentially growing cells were measured. Therefore, cells were harvested from shaker flasks 72 h after inoculation. After a washing step in 0.9% NaCl, dry cell pellets were frozen at -80°C and stored until analysis. Enzyme extraction and assays were performed as described for MDCK cells [46]. Three assay parameters had to be optimized before measuring CR.pIX cell extracts: pH, amount of cells that are used and – for GLNase and GS activity assay – incubation time. After these pre-tests, CR.pIX cell extracts were measured with the following modifications: a two-fold higher cell concentration as for MDCK cells was necessary for the GLNase and GS assay and the incubation time of the GLNase assay was set to 30 min instead of 60 min. The assay plates were analyzed in a 96 well plate reader (Tecan) and enzyme activities were calculated from absorption values.

### Metabolic network

A stoichiometric network model of the central metabolism of avian cells (Figure 1, Table 2) was constructed based on KEGG database entries from *Gallus gallus* (chicken), *Meleagris gallopavo* (turkey) and *Taeniopygia guttata* (zebra finch) as well as on information available from previously published networks and from studies on avian metabolism [40]. Overall, the network consists of 97 reactions and 72 metabolites, resulting in a stoichiometric matrix with dimensions 72 × 97 and 28 degrees of freedom. All implemented reactions are listed in the Table 2.

**Table 2:** Reactions included in the metabolic network model of CR.pIX cells

**Uptake rates**	
r1, Glc	Glc → Glc_cyt_
r2, Pyr	Pyr + 0.33 ATP_cyt_ → Pyr_cyt_
r3, O2	O_2_ → O_2,cyt_
r4, Gln	Gln + 0.33 ATP_cyt_ → Gln_cyt_
r5, Glu	Glu + ATP_cyt_ → Glu_cyt_
r6, Ala	Ala + 0.33 ATP_cyt_ → Ala_cyt_
r7, Asp	Asp + ATP_cyt_ → Asp_cyt_
r8, Arg	Arg + 0.33 ATP_cyt_ → Arg_cyt_
r9, Asn	Asn + 0.33 ATP_cyt_ → Asn_cyt_
r10, Cys	Cys + 0.33 ATP_cyt_ → Cys_cyt_
r11, Gly	Gly + 0.33 ATP_cyt_ → Gly_cyt_
r12, His	His + 0.33 ATP_cyt_ → His_cyt_
r13, Ile	Ile + 0.33 ATP_cyt_ → Ile_cyt_
r14, Leu	Leu + 0.33 ATP_cyt_ → Leu_cyt_
r15, Lys	Lys + 0.33 ATP_cyt_ → Lys_cyt_
r16, Val	Val + 0.33 ATP_cyt_ → Val_cyt_
r17, Met	Met + 0.33 ATP_cyt_ → Met_cyt_
r18, Phe	Phe + 0.33 ATP_cyt_ → Phe_cyt_
r19, Pro	Pro + 0.33 ATP_cyt_ → Pro_cyt_
r20, Ser	Ser + 0.33 ATP_cyt_ → Ser_cyt_
r21, Thr	Thr + 0.33 ATP_cyt_ → Thr_cyt_
r22, Trp	Trp + 0.33 ATP_cyt_ → Trp_cyt_
r23, Tyr	Tyr + 0.33 ATP_cyt_ → Tyr_cyt_
**Glycolysis**	
r24, G6P	Glc_cyt_ + ATP_cyt_ → G6P_cyt_
r25, F6P	G6P_cyt_ ↔ F6P_cyt_
r26, FBP	F6P_cyt_ + ATP_cyt_ ↔ FBP_cyt_
r27, DHAP	FBP_cyt_ ↔ GAP_cyt_ + DHAP_cyt_
r28, GAP	DHAP_cyt_ ↔ GAP_cyt_
r29, PG	GAP_cyt_ ↔ NADH_cyt_ + ATP_cyt_ + PG_cyt_
r30, PEP	PG_cyt_↔ PEP_cyt_
r31, PEP_Pyr	PEP_cyt_ → Pyr_cyt_ + ATP_cyt_
r32, Pyr_Lac	Pyr_cyt_ + NADH_cyt_ ↔ Lac_cyt_
r33, PDH	Pyr_mit_ + CoA_mit_ → AcCoA_mit_ + CO_2,mit_ + NADH_mit_
**Pentose phosphate pathway**	
r34, R5P	G6P_cyt_ → R5P_cyt_ + CO_2,cyt_ + 2 NADPH_cyt_
**TCA cycle**	
r35, OAA	Mal_cyt_ ↔ OAA_cyt_ + NADH_cyt_
r36, Cit	AcCoA_cyt_ + OAA_cyt_ → Cit_cyt_ + CoA_cyt_
r37, Fum_Mal	Fum_cyt_ ↔ Mal_cyt_
r38, Cit_mito_	AcCoA_mit_ + OAA_mit_ → Cit_mit_ + CoA_mit_
r39, OAA_mito_	Mal_mit_ ↔ OAA_mit_ + NADH_mit_
r40, Fum_Mal_mito_	Fum_mit_ ↔ Mal_mit_
r41, SCoA_mito_	aKG_mit_ + CoA_mit_ → SuccCoA_mit_ + CO_2,mit_ + NADH_mit_
r42, Fum_mito_	SuccCoA_mit_ ↔ Fum_mit_ + CoA_mit_ + ATP_mit_ + FADH_2,mit_
r43, aKG_mito_	Cit_mit_ ↔ aKG_mit_ + CO_2,mit_ + NADH_mit_
**Anaplerosis**	
r44, Ana_PyrI	Mal_cyt_ → Pyr_cyt_ + CO_2,cyt_ + NADPH_cyt_
r45, Ana_PyrII	Mal_mit_ ↔ Pyr_mit_ + CO_2,mit_ + NADPH_mit_
r46, PC	Pyr_mit_ + CO_2,mit_ + ATP_mit_ → OAA_mit_
**Amino acid catabolism**	
r47, GDH	Glu_mit_ ↔ aKG_mit_ + Amm_mit_ + NADPH_mit_
r48, GS	Gln_cyt_ ↔ Glu_cyt_ + Amm_cyt_ + ATP_cyt_
r49, Ala_cat_	Ala_cyt_ + aKG_cyt_ ↔ Pyr_cyt_ + Glu_cyt_
r50, Asn_Asp	Asn_cyt_ ↔ Asp_cyt_ + Amm_cyt_
r51, His_cat_	His_cyt_ → Glu_cyt_ + 2 Amm_cyt_ + CO_2,cyt_
r52, Ile_cat_	Ile_cyt_ + aKG_cyt_ + 2 CoA_mit_ + ATP_mit_ → SuccCoA_mit_ + AcCoA_mit_ + Glu_cyt_ + NADH_mit_ + FADH_2,mit_
r53, Leu_cat_	Leu_cyt_ + aKG_cyt_ + 3 CoA_mit_ + 2 ATP_mit_ → 3 AcCoA_mit_ + Glu_cyt_ + NADH_mit_ + FADH_2,mit_
r54, Lys_cat_	Lys_cyt_ + 2 aKG_cyt_ + 2 CoA_mit_ + NADPH_cyt_ → 2 AcCoA_mit_ + 2 Glu_cyt_ + 2 CO_2,mit_ + 2 NADH_cyt_ + 2 NADH_mit_ + FADH_2,mit_
r55, Met_cat_	Met_cyt_ + Ser_cyt_ + CoA_mit_ + 3 ATP_cyt_ + ATP_mit_ → Cys_cyt_ + SuccCoA_mit_ + Amm_cyt_ + NADH_mit_ + CO_2,cyt_
r56, Phe_cat_	Phe_cyt_ + O_2,cyt_ + NADPH_cyt_ → Tyr_cyt_
r57, Pro_cat_	Pro_cyt_ ↔ Glu_cyt_ + 2 NADH_cyt_
r58, Thr_cat_	Thr_cyt_ → Pyr_mit_ + Amm_mit_ + CO_2,mit_ + NADH_cyt_ + NADH_mit_ + FADH_2,mit_
r59, Trp_cat_	Trp_cyt_ + 2 CoA_mit_ + 3 O_2,cyt_ + NADPH_cyt_ → Ala_cyt_ + 2 AcCoA_mit_ + Amm_cyt_ + 2 CO_2,mit_ + 2 CO_2,cyt_ + NADH_cyt_ + 2 NADH_mit_ + FADH_2,mit_
r60, Val_cat_	Val_cyt_ + aKG_cyt_ + CoA_mit_ + ATP_mit_ → SuccCoA_mit_ + Glu_cyt_ + CO_2,cyt_ + 3 NADH_mit_ + FADH_2,mit_
r61, Tyr_cat_	Tyr_cyt_ + aKG_cyt_ + 2 CoA_cyt_ + 2 O_2,cyt_ → Fum_cyt_ + 2 AcCoA_cyt_ + Glu_cyt_ + CO_2,cyt_
r62, Ser_cat_	Ser_cyt_ ↔ Pyr_cyt_ + Amm_cyt_
r63, Cys_cat_	Cys_cyt_ + aKG_cyt_ + O_2,cyt_ → Pyr_cyt_ + Glu_cyt_
r64, Asp_cat_	Asp_cyt_ + aKG_mit_ ↔ Glu_cyt_ + OAA_mit_
r65, Arg_cat_	Arg_cyt_ + aKG_cyt_ → 2 Glu_cyt_ + Urea_cyt_ + NADH_cyt_
**MTHF & uric acid synthesis**	
r66, MTHF_I	Ser_cyt_ + THF_cyt_ → Gly_cyt_ + MTHF_cyt_
r67, MTHF_II	Gly_cyt_ + THF_cyt_ → NADH_cyt_ + CO_2,cyt_ + Amm_cyt_ + MTHF_cyt_
r68, UricAcid	Asp_cyt_ + 2 Gln_cyt_ + Gly_cyt_ + 2 MTHF_cyt_ + 7 ATP_cyt_ + CO_2,cyt_ → UricAcid_cyt_ + Fum_cyt_ + 2 Glu_cyt_ + 2 THF_cyt_
**Lipid synthesis**	
r69, CH_Lip_	18 AcCoA_cyt_ + 18 ATP_cyt_ + 11 O_2,cyt_ + 27 NADPH_cyt_ → CH + 18 CoA_cyt_ + 9 CO_2,cyt_
r70, PC_Lip_	GAP_cyt_ + Ser_cyt_ + 27.6 ATP_cyt_ + 17.6 AcCoA_cyt_ + 4 MTHF_cyt_ + 2 NADH_cyt_ + 31.2 NADPH_cyt_ → PC + 17.6 CoA_cyt_ + 4 THF_cyt_
r71, PE_Lip_	GAP_cyt_ + Ser_cyt_ + 18.6 ATP_cyt_ + 17.6 AcCoA_cyt_ + MTHF_cyt_ + 2 NADH_cyt_ + 31.2 NADPH_cyt_ → PE + 17.6 CoA_cyt_ + THF_cyt_
r72, PS_Lip_	GAP_cyt_ + Ser_cyt_ + 18.6 ATP_cyt_ + 17.6 AcCoA_cyt_ + 2 MTHF_cyt_ + 2 NADH_cyt_ + 31.2 NADPH_cyt_ → PS + 17.6 CoA_cyt_ + 2 THF_cyt_
r73, PGL_Lip_	2 GAP_cyt_ + 17.6 ATP_cyt_ + 17.6 AcCoA_cyt_ + 4 NADH_cyt_ + 31.2 NADPH_cyt_ → PGL + 17.6 CoA_cyt_
r74, PI_Lip_	GAP_cyt_ + G6P_cyt_ + 17.6 ATP_cyt_ + 17.6 AcCoA_cyt_ + 2 NADH_cyt_ + 31.2 NADPH_cyt_ → PI + 17.6 CoA_cyt_
r75, SM_Lip_	2 Ser_cyt_ + 27.8 ATP_cyt_ + 16.8 AcCoA_cyt_ + 3 MTHF_cyt_ + 2 NADH_cyt_ + 29.6 NADPH_cyt_ → SM + 16.8 CoA_cyt_ + 3 THF_cyt_
r76, DPG_Lip_	3 GAP_cyt_ + 35.2 ATP_cyt_ + 35.2 AcCoA_cyt_ + 6 NADH_cyt_ + 62.4 NADPH_cyt_ → DPG + 35.2 CoA_cyt_
**Release rates**	
r77, ATP_main_	ATP_cyt_ → maintenance
r78, Lac_out_	Lac_cyt_ → Lac
r79, Ala_out_	Ala_cyt_ → Ala
r80, UricAcid_out_	Uric acid_cyt_ → Uric acid
r81, Urea_out_	Urea_cyt_ → Urea
r82, Amm_out_	Amm_cyt_ → Amm
r83, CO_2 out_	CO_2,cyt_ → CO_2_
r84, Pyr_out_	Pyr_cyt_ → Pyr
**Transport reactions, oxidative phosphorylation**	
r85, NADH_cyt,trans_	NADH_cyt_ ↔ NADH_mit_
r86, ATP_trans_	ATP_cyt_ ↔ ATP_mitt_
r87, CO_2 trans_	CO_2,cyt_ ↔ CO_2,mit_
r88, MAL_trans_	Mal_cyt_ + Cit_mit_ ↔ Mal_mit_ + Cit_cyt_
r89, Glu_trans_	Glu_cyt_ ↔ Glu_mit_
r90, Pyr_trans_	Pyr_cyt_ ↔ Pyr_mit_
r91, aKG_trans_	aKG_cyt_ ↔ aKG_mit_
r92, Amm_trans_	Amm_cyt_ ↔ Amm_mit_
r93, FADH_ox_	O_2,cyt_ + 2 FADH_2,mit_ → 3 ATP_mit_
r94, NADH_mit,trans_	NADH_mit_ ↔ NADPH_mit_
r95, NADH_ox_	O_2,cyt_ + 2 NADH_mit_ → 5 ATP_mit_
r96, NADH_cyt,trans_	NADH_cyt_ ↔ NADPH_cyt_
**Synthesis of macromolecules and biomass**	
r97, μ	0.552 proteins + 0.263 carbohydrates + 0.131 lipids + 0.023 DNA + 0.031 RNA → biomass
Proteins [1 g] = 955.79 Asp_cyt_ + 1344.29 Ala_cyt_ + 543.16 Gln_cyt_ + + 817.99 Glu_cyt_ + 1024.72 Arg_cyt_ + 362.10 Asn_cyt_ + 19.61 Cys_cyt_ + 913.16 Gly_cyt_ + 271.58 His_cyt_ + 240.54 Ile_cyt_ + 588.41 Leu_cyt_ + 724.21 Lys_cyt_ + 191.97 Val_cyt_ + 114.57 Met_cyt_ + 201.91 Phe_cyt_ + 205.97 Pro_cyt_ + 247.44 Ser_cyt_ + 202.07 Thr_cyt_ + 21.17 Trp_cyt_ + 61.87 Tyr_cyt_ + 24046.3 ATP_cyt_	
Lipids [1 g] = 181 CH + 661.4 PC + 250.3 PE + 90.9 PI + 24.9 PS + 12.6 PGL + 81.4 SM + 26.8 DPG	
DNA [1 g] = 3009 R5P_cyt_ + 3912 Asp_cyt_ + 5717 Gln_cyt_ + -2106 NADH_cyt_ + 1505 Gly_cyt_ + 22569 ATP_cyt_ + 903 NADH_mit_ + -2407 Mal_cyt_ + 903 NADPH_cyt_ + -5717 Glu_cyt_ + 5417 MTHF_cyt_ + -5417 THF_cyt_	
RNA [1 g] = 3020 R5P_cyt_ + 3606 Asp_cyt_ + 6316 Gln_cyt_ + 293 O_2,cyt_ + -2435 NADH_cyt_ + 1477 Gly_cyt_ + 22614 ATP_cyt_ + 586 NADH_mit_ + -2069 Mal_cyt_ + -2954 NADPH_cyt_ + -6316 Glu_cyt_ + 4431 MTHF_cyt_ + -4431 THF_cyt_	
Carbohydrates [1 g] = 6172.8 G6P_cyt_ + 21605 ATP_cyt_	

Generally, the network includes

•Two compartments: cytosol and mitochondria

•Main pathways of energy metabolism: glycolysis, TCA cycle and oxidative phosphorylation

•Catabolism of amino acids

•Transport of metabolites and corresponding energy demands (as described before [14])

•Synthesis of macromolecules (biomass)

•Synthesis of urea and uric acid

•A simplified/lumped pentose-phosphate pathway (as in [9])

### Calculation of extracellular rates

During the exponential growth phase, metabolism is considered to be in a pseudo steady-state with constant reaction rates [55]. The growth rate μ was calculated via curve fitting of the viable cell concentration measurements (X(t)) to:

(1) $$X\left(t\right)=X\left(t_{0}\right)\cdot e^{\mu \cdot \left(t-t_{0}\right)}$$

Uptake or release rates *v*_*i*_ (in unit [μmol/gDW/h] of external metabolites *c*_*i*_ were computed via curve fitting of the concentration data to:

(2) $$c_{i}\left(t\right)=c_{i}\left(t_{o}\right)+\nu_{i}\cdot \frac{\mathrm{DW}\left(t_{0}\right)}{\mu }\cdot \left(e^{\mu \cdot \left(t-t_{0}\right)}-1\right),$$

where we applied the experimentally determined cell dry weight and the calculated *μ* from Equation (1).

For glutamine, a first order degradation process to ammonia and 5-oxopyrrolidine-2-carboxylic acid was assumed. The rate constant *k*_*Gln*_ = 0.0032 h^-1^ of this process was determined experimentally (n = 3) using medium without cells under standard cultivation conditions in a bioreactor. Based on this degradation rate, glutamine and ammonia rates (Equation (2)) were corrected as described elsewhere [56]. To compute standard deviations of the fluxes, we performed a Monte-Carlo simulation and added a normally distributed error with mean zero and corresponding standard deviation (derived from assay validations) at each time point and for each measurement (metabolites and cell concentration). With this, we generated 100,000 random sample data sets and recalculated for each data set the rates of uptake, release and growth. From these simulated data sets, the empirical variances and standard deviations $\sigma_{r_{i}}$ for each of these rates were computed.

### Flux variability and metabolic flux analysis

We restrict our analysis to the time span of exponential growth from 24 h to 97 h. Within this time interval the quasi steady state assumption holds and, together with the stoichiometric matrix **N** and the vector **r** of reaction rates (fluxes), the reaction rates can be determined by solving a system of linear equations. Given the measured and known rates, **r** and **N** can be partitioned into the known (**r**_**k**_, **N**_**k**_) and unknown part (**r**_**u**_, **N**_**u**_) we can write the steady state equation as

$$\frac{\mathrm{dc}}{\mathrm{dt}}=0=N\cdot r=N_{u}r_{u}+N_{k}r_{k}$$

yielding after reformulation the central equation for metabolic flux analysis

(3) $$N_{u}r_{u}=-N_{k}r_{k}.$$

A detailed description of how to further proceed with Equation (3) is given elsewhere [57-59]. If the linear equation system is over-determined, a variance-weighted least squares estimation can be applied to calculate uniquely determined reaction rates. However, Equation (3) usually defines an underdetermined system and, hence, the flux distribution cannot be calculated uniquely. For identifying at least feasible ranges of the fluxes we use flux variability analysis (FVA), an approach that is based on linear programming [27]. Here, the minimal and maximal possible flux that a reaction can have in any flux distribution that is consistent with Equation (3) is calculated separately for each unknown reaction *r*_*u*,*i*_,. Further constraints arise by the irreversibility of some reactions (constrained to non-negative values) or because some reaction rates are fixed to specific (measured or known) values reflecting the given environmental conditions. To avoid unbounded fluxes and to reflect thermodynamic limits under physiological conditions, upper bounds of unknown reaction rates were set to 10000 μmol/gDW/h. The optimization problems studied by FVA then read

(4) $$\begin{array}{c} \begin{array}{ccc} r_{u,i}^{\text{min}}=\min_{r}r_{u,i} & \mathrm{OR} & r_{u,i}^{\text{max}}=\max_{r}r_{u,i} \end{array}\\ s.t.\\ N_{u}r_{u}=-N_{k}r_{k}\\ \begin{array}{ccccc} 0\leq r_{i}\leq 10000 & \forall i & \mathrm{reaction} & i & \mathrm{irreversible} \end{array}\\ \begin{array}{ccccc} -10000\leq r_{i}\leq 10000 & \forall i & \mathrm{reaction} & i & \mathrm{reversible} \end{array} \end{array}$$

As a result from the 2∗*u* optimizations (*u* = number of unknown fluxes), we obtained the physiologically feasible flux range for the unknown reactions. Moreover, if the computed minimal and maximal rate of a reaction coincide (*r*_*u*,*i*_^min^ = *r*_*u*,*i*_^max^), the reaction rate follows to be uniquely determined. Note that FVA as described above does not make any assumption about biological objectives in contrast to flux balance analysis [59]. The objective function in equation (4) only serves as a tool to identify the feasible flux ranges. This approach has also been used to estimate flux distributions in CHO cells and was introduced under the term flux-spectrum [60].

We define two scenarios where we consider different sets of constraints. In scenario 1, we applied the measured rate values plus/minus their corresponding standard deviations as upper/lower boundaries for the corresponding rates. In case that the lower boundary of an uptake rate becomes negative, we set it to zero. Rates that were measured not to be active (e.g. uric acid excretion and alanine uptake) were also fixed to zero. No further constraints were used so that the results obtained with this scenario are robust against measurement errors and assumptions on reaction activities. However, since the system is underdetermined with these constraints, only flux ranges can be calculated via FVA.

In scenario 2, we set the measured uptake/excretion rates as fixed constraints. To resolve the anaplerotic fluxes and since the corresponding flux range contains zero (see Results), we assumed the pyruvate-carboxylase (r46) to be inactive. This assumption is biologically reasonable as it is often described and assumed to be negligible in transformed cell cultures (e.g. in [25,61]) and supported by our enzyme measurements. With these constraints the network is over-determined with two degrees of redundancy. By applying MFA with a variance-weighted least squares estimation [57], we can calculate a unique flux distribution. To avoid a significant deviation of the estimated against the measured growth rate, we set the measurement variance of the growth rate to 1 × 10^-9^ in scenario 2.

All computations presented in this study were performed with our software *CellNetAnalyzer*, a MATLAB toolbox with graphical user interface facilitating metabolic network analysis [28]. It can be downloaded from http://www.mpi-magdeburg.mpg.de/projects/cna/cna.html, and the network project files will be made available on this site within *CellNetAnalyzer*’s model repository.

## Competing interests

Patent applications covering avian cell lines (including AGE1.CR.pIX) have been filed by I. Jordan.

## Authors’ contributions

VL and OH conceived the experiments or calculations, interpreted the data and drafted the manuscript. IJ and YG edited the manuscript and participated in planning the experiments. HB helped in data analyses and manuscript drafting. SK contributed to the conception of the study and edited the manuscript. UR helped in drafting the manuscript and generally supervised the project. All authors read and approved the final manuscript.

## Supplementary Material

Additional file 1 **Table S1.** Amino acid composition of cell protein from CR.pIX cells and other cell lines. **Table S2.** Calculated rate ranges (scenario 1; by FVA) or rates (scenario 2; by MFA) for CR.pIX cells cultured in stirred tank reactors. **Table S3.** Measured concentrations, standard deviations of validated assays, and measurement devices. Click here for file
