# Supplementary material for: The avian cell line AGE1.CR.pIX characterized by metabolic flux analysis
Source: BMC Biotechnol. 2014 Jul 30;14:72. doi: 10.1186/1472-6750-14-72 (PMC4124504; doi:10.1186/1472-6750-14-72)
Supplement: Additional file 1 — Table S1. Amino acid composition of cell protein from CR.pIX cells and other cell lines. Table S2. Calculated rate ranges (scenario 1; by FVA) or rates (scenario 2; by MFA) for CR.pIX cells cultured in stirred tank reactors. Table S3. Measured concentrations, standard deviations of validated assays, and measurement devices. [file 1472-6750-14-72-S1.docx]

**Table S1:** Amino acid composition of cell protein from CR.pIX cells and other cell lines.

| **Amino acid** | **CR.pIX** | **Mouse hybridoma^a^** | **Sf9^b^** | **AGE1.hn^c^** | **CHO^d^** | ***Pichia pastoris*^e^** |
| --- | --- | --- | --- | --- | --- | --- |
| Ala | 16.3 ± 1.1^f^ | 8.3 | 7.0 | 8.1 | 8.7 | 10.7 |
| Arg | 11.2 ± 2.1 | 5.9 | 8.0 | 5.2 | 4.9 | 6.7 |
| Asn | 4.5^g^ | 4.4 | 5.9 | 9.3^h^ | 5.4 | 8.8^h^ |
| Asp | 5.5 ± 0.4 | 4.7 | 5.9 |  | 4.3 |  |
| Cys | 2.0^g^ | 2.8 | - | 2.8^g^ | 1.8 | ~0 |
| Glu | 4.7 ± 0.3 | 5.0 | 7.0 | 12.8^h^ | 4.9 | 18.6^h^ |
| Gln | 6.0^g^ | 6.2 | 7.0 |  | 5.9 |  |
| Gly | 11.1 ± 0.8 | 8.5 | 5.4 | 9.5 | 8.1 | 7.1 |
| His | 3.0^g^ | 2.2 | 3.1 | 2.2 | 2.0 | 1.8 |
| Ile | 2.9 | 4.2 | 5.9 | 4.5 | 4.8 | 4.1 |
| Leu | 7.4 ± 0.4 | 8.1 | 8.7 | 8.7 | 8.5 | 7.0 |
| Lys | 8.0^g^ | 6.8 | 9 | 7.5 | 8.5 | 6.3 |
| Met | 1.4 ± 0.3 | 2.2 | 3.7 | 1.7^g^ | 2.2 | 0.8 |
| Phe | 2.5 ± 0.3 | 3.2 | 5.5 | 3.6 | 3.5 | 3.0 |
| Pro | 2.2 ± 0.2 | 5.3 | 5.3 | 4.0 | 5.3 | 3.7 |
| Ser | 3.3 ± 0.3 | 6.9 | 4.6 | 6.2 | 6.3 | 6.4 |
| Thr | 2.5 ± 0.4 | 5.7 | 4.8 | 4.9 | 5.5 | 5.8 |
| Trp | 2.0^g^ | 1.1 | 2.6 | 1.1^g^ | 0.6 | 1.4 |
| Tyr | 3.0 ± 0.9 | 2.6 | 4.6 | 1.9 | 2.6 | 2.1 |
| Val | 2.3 ± 0.2 | 6.0 | 6.6 | 6.1 | 6.1 | 5.6 |

^a^from ([Xie and Wang, 1994a](#_ENREF_37)); ^b^from ([Ferrance et al., 1993](#_ENREF_12)); ^c^from ([Niklas et al., 2011](#_ENREF_26)); ^d^from ([Selvarasu et al., 2012](#_ENREF_30)) where average values for CHO were taken; ^e^from ([Carnicer et al., 2009](#_ENREF_9))

^f^standard deviation from n=5 measurements

^g^values estimated from literature as these amino acids could either not be measured or were decomposed during acidic hydrolysis of cell protein

^h^glutamate/glutamine and aspartate/asparagine are given as a sum as the deamination during protein hydrolysis was not calculated

**Table S2**: Calculated rate ranges (scenario 1; by FVA) or rates (scenario 2; by MFA) for CR.pIX cells cultured in stirred tank reactors. Values in italics indicate fluxes that have been measured. All fluxes are given in [µmol/gDW/h].

| **Reaction** | **Scenario 1** | | | | **Scenario 2** | | |  | **Reaction** | **Scenario 1** | | | | **Scenario 2** | | |
| --- | --- | --- | --- | --- | --- | --- | --- | --- | --- | --- | --- | --- | --- | --- | --- | --- |
| **Uptake rates** | | | | | | | |  | **Amino acid catabolism** | | | | | | | |
| r1, Glc | | *200.42-222.05* | | | | *211.24* | |  | r47, GDH | | -22.56-23.25 | | | | -3.16 | |
| r2, Pyr | | *29.39-30.96* | | | | *30.18* | |  | r48, GS | | -8.39-(-8.10) | | | | -8.26 | |
| r3, O2 | | 54.43-450.10 | | | | 230.41 | |  | r49, Ala_cat_ | | -47.13-(-36.73) | | | | -41.25 | |
| r4, Gln | | *0.00-0.10* | | | | *0.00* | |  | r50, Asn_Asp | | 5.58-22.62 | | | | 19.03 | |
| r5, Glu | | *0.33-2.43* | | | | *1.42* | |  | r51, His_cat_ | | 0.00-0.77 | | | | 0.09 | |
| r6, Ala | | *0.00-0.00* | | | | *0.00* | |  | r52, Ile_cat_ | | 0.00-8.77 | | | | 4.70 | |
| r7, Asp | | *14.52-20.99* | | | | *18.12* | |  | r53, Leu_cat_ | | 0.00-6.50 | | | | 2.63 | |
| r8, Arg | | *7.16-12.74* | | | | *7.22* | |  | r54, Lys_cat_ | | 0.00-0.54 | | | | 0.62 | |
| r9, Asn | | *8.58-25.56* | | | | *21.99* | |  | r55, Met_cat_ | | 1.31-5.21 | | | | 3.35 | |
| r10, Cys | | 0.00-0.00 | | | | 0.00 | |  | r56, Phe_cat_ | | 0.00-1.97 | | | | 0.58 | |
| r11, Gly | | *0.00-9.53* | | | | *5.08* | |  | r57, Pro_cat_ | | -1.43-(-1.30) | | | | -1.41 | |
| r12, His | | *1.96-2.72* | | | | *2.06* | |  | r58, Thr_cat_ | | 0.00-3.73 | | | | 1.32 | |
| r13, Ile | | *1.86-10.63* | | | | *6.57* | |  | r59, Trp_cat_ | | 0.00-0.39 | | | | 0.01 | |
| r14, Leu | | *4.69-11.19* | | | | *7.35* | |  | r60, Val_cat_ | | 0.00-5.67 | | | | 2.90 | |
| r15, Lys | | *5.22-5.76* | | | | *5.88* | |  | r61, Tyr_cat_ | | 0.00-3.63 | | | | 0.49 | |
| r16, Val | | *1.48-7.15* | | | | *4.39* | |  | r62, Ser_cat_ | | -17.98-7.62 | | | | -2.81 | |
| r17, Met | | *2.19-6.10* | | | | *4.25* | |  | r63, Cys_cat_ | | 0.00-3.91 | | | | 2.04 | |
| r18, Phe | | *1.56-3.53* | | | | *2.15* | |  | r64, Asp_cat_ | | -13.80-37.46 | | | | 30.95 | |
| r19, Pro | | *0.00-0.10* | | | | *0.00* | |  | r65, Arg_cat_ | | 0.00-5.58 | | | | 0.01 | |
| r20, Ser | | *0.00-16.66* | | | | *10.59* | |  | **MTHF & uric acid synthesis** | | | | | | | |
| r21, Thr | | *1.56-5.29* | | | | *2.90* | |  | r66, MTHF_I | | 3.74-8.70 | | | | | 6.03 |
| r22, Trp | | *1.31-1.70* | | | | *1.33* | |  | r67, MTHF_II | | 0.40-5.17 | | | | | 2.94 |
| r23, Tyr | | *0.00-3.62* | | | | *1.89* | |  | r68, UricAcid | | 0.00-0.00 | | | | | 0.00 |
| **Glycolysis** | | | | | | | |  | **Lipid synthesis** | | | | | | | |
| r24, G6P | | 200.42-222.05 | | | | | 211.24 |  | r69, CH_Lip_ | 0.31-0.32 | | | | 0.31 | | |
| r25, F6P | | 176.32-198.49 | | | | | 187.51 |  | r70, PC_Lip_ | 1.14-1.16 | | | | 1.14 | | |
| r26, FBP | | 176.32-198.49 | | | | | 187.51 |  | r71, PE_Lip_ | 0.43-0.44 | | | | 0.43 | | |
| r27, DHAP | | 176.32-198.49 | | | | | 187.51 |  | r72, PS_Lip_ | 0.043-0.044 | | | | 0.043 | | |
| r28, GAP | | 176.32-198.49 | | | | | 187.51 |  | r73, PGL_Lip_ | 0.021-0.022 | | | | 0.022 | | |
| r29, PG | | 350.66-395.04 | | | | | 373.07 |  | r74, PI_Lip_ | 0.156-0.160 | | | | 0.157 | | |
| r30, PEP | | 350.66-395.04 | | | | | 373.07 |  | r75, SM_Lip_ | 0.140-0.143 | | | | 0.141 | | |
| r31, PEP_Pyr | | 350.66-395.04 | | | | | 373.07 |  | r76, DPG_Lip_ | 0.046-0.047 | | | | 0.046 | | |
| r32, Pyr_Lac | | 288.82-306.39 | | | | | 297.61 |  | **Release rates** | | | | | | | |
| r33, PDH | | 53.08-150.33 | | | | | 102.04 |  | r77, ATP_main_ | | | 0.00-1996.42 | 965.79 | | | |
| **Pentose phosphate pathway** | | | | | | | |  | r78, Lac_out_ | | | *288.82-306.39* | *297.61* | | | |
| r34, R5P | 2.13-2.18 | | | | 2.15 | | |  | r79, Ala_out_ | | | *26.73-36.50* | *30.81* | | | |
| **TCA cycle** | | | | | | | |  | r80, UricAcid_out_ | | | *0.00-0.00* | *0.00* | | | |
| r35, OAA | | | | -41.91-(33.71) | | | -40.28 |  | r81, Urea_out_ | | | 0.00-5.58 | 0.01 | | | |
| r36, Cit | | | | -41.91-(-33.71) | | | -40.28 |  | r82, Amm_out_ | | | *11.70-13.56* | *12.60* | | | |
| r37, Fum_Mal | | | | 0.00-3.63 | | | 0.49 |  | r83, CO_2 out_ | | | 140.29-492.32 | 300.87 | | | |
| r38, Cit_mito_ | | | | 60.67-176.63 | | | 115.90 |  | r84, Pyr_out_ | | | *0.00-0.00* | *0.00* | | | |
| r39, OAA_mito_ | | | | -1845.84-159.43 | | | 84.95 |  | **Transport reactions, oxidative phosphorylation** | | | | | | | |
| r40, Fum_Mal_mito_ | | | | 18.15-156.79 | | | 79.70 |  | r85, NADH_trans_ | | | -70.43-19.72 | | -30.19 | | |
| r41, SCoA_mito_ | | | | 16.82-137.13 | | | 68.75 |  | r86, ATP_trans_ | | | 81.69-2102.95 | | 1087.58 | | |
| r42, Fum_mito_ | | | | 18.15-156.79 | | | 79.70 |  | r87, CO_2 trans_ | | | -466.50-(-126.43) | | -284.04 | | |
| r43, aKG_mito_ | | | | 18.76-142.93 | | | 75.62 |  | r88, Mal_trans_ | | | 41.91-33.71 | | 40.28 | | |
| **Anaplerosis** | | | | | | | |  | r89, Glu_trans_ | | | -22.56-23.25 | | -3.16 | | |
| r44, Ana_PyrI | | | 1.57-5.20 | | | | 2.07 |  | r90, Pyr_trans_ | | | 14.01-113.28 | | 65.69 | | |
| r45, Ana_PyrII | | | 14.72-2036.34 | | | | 35.03 |  | r91, aKG_trans_ | | | -47.13-(-1.59) | | -27.24 | | |
| r46, PC | | | 0.00-1996.42 | | | | 0.00 |  | r92, Amm_trans_ | | | -25.12-18.83 | | 1.84 | | |
|  | | | | | | | |  | r93, FADH_ox_ | | | 9.08-91.19 | | 45.94 | | |
|  |  | | | |  | | |  | r94, NADH_mit,trans_ | | | -2055.10-(-2.61) | | -31.87 | | |
|  |  | | | |  | | |  | r95, NADH_ox_ | | | 40.77-341.05 | | 177.25 | | |
|  |  | | | |  | | |  | r96, NADH_cyt,trans_ | | | 62.66-68.51 | | 65.51 | | |
|  |  | | | |  | | |  | **Biomass synthesis** | | | | | | | |
|  |  | | | |  | | |  | r97, µ | 0.0131-0.0134 | | | 0.0132 | | | |

Table S3: Overview: measured concentrations, standard deviations of validated assays, and measurement devices.

| **Measured concentrations** | **Standard deviation**  **of the method** | **Device** |
| --- | --- | --- |
| Cell | 2.5 %^a^ | ViCELL XR (BeckmanCoulter) |
| Glucose | 0.39 mM^b^ | BioProfile 100plus  (Nova Biomedicals) |
| Lactate | 0.30 mM^b^ |  |
| Ammonium | 4.5 %^a^ |  |
| Pyruvate | 2.1 %^a^ | HPLC (DX-320, Dionex) |
| Alanine | 8.6 %^a^/5 %^c^ | HPLC (ICS-5000, Dionex)/  RP-HPLC (Kontron D450) |
| Arginine | 3.2 %^a^/5 %^c^ |  |
| Asparagine | 0.44 mM^b^/5 %^c^ |  |
| Aspartate | 0.55 mM^b^/5 %^c^ |  |
| Cysteine | 0.09 mM^b^/5 %^c^ |  |
| Glutamate | 0.03 mM^b^/5 %^c^ |  |
| Glutamine | 12.8 %^a^/5 %^c^ |  |
| Glycine | 2.7 %^a^/5 %^c^ |  |
| Histidine | 0.54 mM^b^/5 %^c^ |  |
| Isoleucine | 0.64 mM^b^/5 %^c^ |  |
| Leucine | 0.56 mM^b^/5 %^c^ |  |
| Methionine | 0.33 mM^b^/5 %^c^ |  |
| Phenylalanine | 0.13 mM^b^/5 %^c^ |  |
| Threonine | 0.11 mM^b^/5 %^c^ |  |
| Tryptophan | 0.22 mM^b^/5 %^c^ |  |
| Tyrosine | 0.32 mM^b^/5 %^c^ |  |
| Valine | 0.82 mM^b^/5 %^c^ |  |

^a^relative standard deviations of the method were taken for those parameters that have shown an inhomogeneity of variances

^b^absolute standard deviations of the method were taken for those parameters that have shown homogeneous variances

^c^extracellular amino acid concentrations for metabolic flux analysis were measured with a derivatization method having a measurement error of 5 %.
